# Differentiating Giant Bullous Emphysema From Tension Pneumothorax: A Case Report

**DOI:** 10.7759/cureus.55988

**Published:** 2024-03-11

**Authors:** Maria Patricia Ascano, Nicholas Kramer, Khoa Le

**Affiliations:** 1 College of Osteopathic Medicine, Touro University Nevada, Henderson, USA; 2 Department of Internal Medicine, North Vista Hospital, Las Vegas, USA

**Keywords:** high-resolution computed tomography (hrct), dyspnea, vanishing lung, giant bullous emphysema, tension pneumothorax, bullous emphysema

## Abstract

Giant bullous emphysema (GBE) is a progressive disease that commonly presents with severe progressive dyspnea attributed to the progressive destruction of alveolar walls and the formation of large air pockets, resulting in impaired gas exchange. This presentation is most commonly seen in young, thin male smokers. GBE poses an interesting and unique clinical challenge due to its radiologic findings, which can be easily mistaken for tension pneumothorax. Despite the decreased acuity of GBE as compared to tension pneumothorax, inadequate treatment in a severe case can lead to spontaneous pneumothorax, infection, and/or respiratory failure. In this report, we highlight a case of severe GBE that presents similarly to tension pneumothorax in both symptomatology and radiologic findings. The case at hand is of a 50-year-old male patient with a history of chronic obstructive pulmonary disease (COPD) with complaints of dyspnea and subsequent findings of tachycardia, tachypnea, and hypoxemia with significantly decreased breath sounds in the right lung. Radiologic findings showed increased lucency of the right hemithorax and a mass effect with a mediastinal shift to the left. History and further imaging with CT led to an ultimate diagnosis of severe GBE and COPD exacerbations. The patient was treated with non-invasive medical management. With the challenges of overlapping presentations, landing on the correct diagnosis is imperative to accurately and adequately treat the patient since GBE and tension pneumothorax significantly differ in acuity and overall management, hence the need for a high level of suspicion based on the clinical picture and the use of high-resolution CT.

## Introduction

Giant bullous emphysema (GBE), or vanishing lung syndrome, is a progressive disease described initially by Burke as characteristically presenting with severe progressive dyspnea with accompanying radiologic findings of extensive and predominantly asymmetric upper lobe bullous emphysema, typically seen in young, thin, male smokers [1]. With the increasing severity and progression of the disease, complications may arise, such as spontaneous pneumothorax, infection, and/or respiratory failure [2].

Many reports have utilized the radiographic criteria proposed by Roberts et al., consisting of: “(1) the presence of giant bullae in one or both upper lobes of the lung; (2) occupying at least one-third of the hemithorax; and (3) compressing the normal surrounding parenchyma” [3]. By juxtaposing multiple cases of GBE, Stern et al. identified that on average, high-resolution computed tomography (HRCT) scans showed that bullae in this category range from 1 to 20 cm in diameter without a single dominant giant bulla, with most ranging from 2 to 8 cm [4].

Several factors have been associated with GBE, such as cigarettes, inhaled drug abuse, alpha-1 antitrypsin deficiency, and connective tissue diseases (e.g., Marfan syndrome and Ehlers-Danlos syndrome), with the most common type related to cigarette use and resultant chronic obstructive pulmonary disease (COPD) [4-5].

## Case presentation

A 50-year-old Caucasian male with a past medical history of COPD, active half-pack per day cigarette use, and moderate amphetamine use presented to the ED with shortness of breath, denying any other medical concerns or complaints. On admission, the patient was found to have tachycardia, tachypnea, and hypoxemia, with room air saturation dropping to 88%. Physical examination revealed little to no breath sounds on the right lobe of the lung, wheezing on the left lobe, and accessory muscle use at rest. The physical examination was otherwise normal.

**Figure 1:**
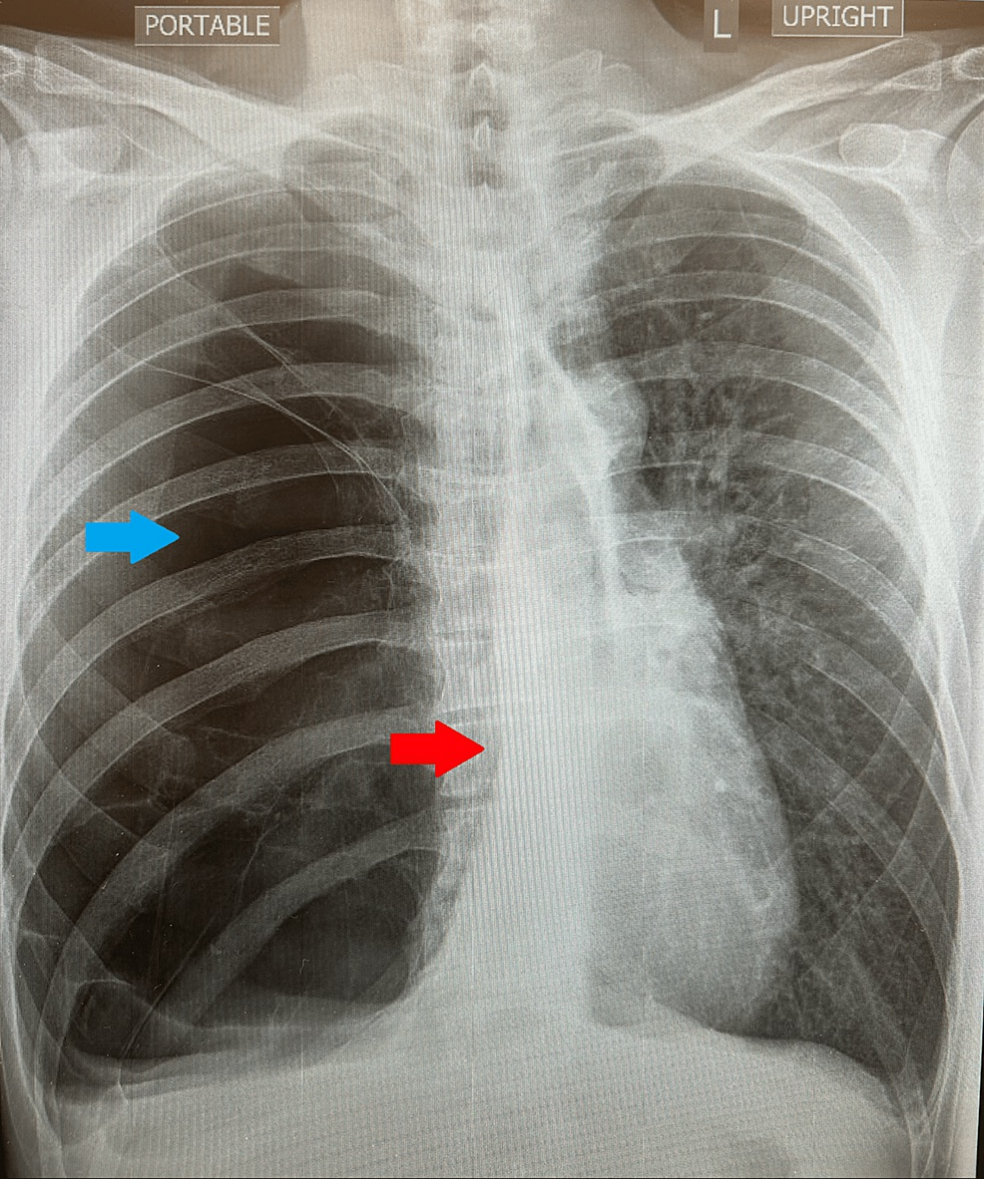
Chest X-ray of the 50-year-old male patient The plain radiograph depicts hyperlucency (blue ➨) of the right hemithorax and a subsequent mass effect causing a left mediastinal shift (red ➨).

**Figure 2:**
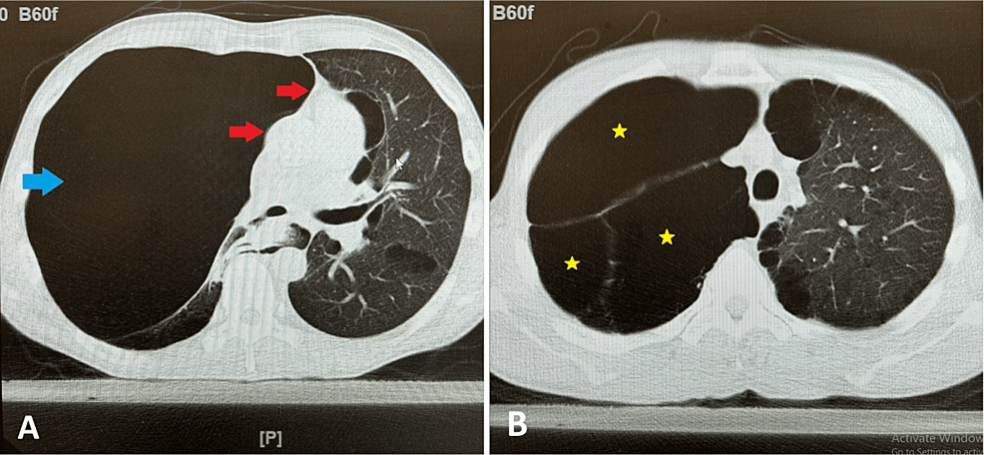
Non-contrast CT of the chest of the same 50-year-old male patient (A) Imaging shows a near-entire right lung involvement (blue ➨) and a mediastinal shift to the left (red ➨). (B) The right lung demonstrates multiple large bullous emphysema adjacent to one another (yellow ★). CT: computed tomography

The chest X-ray depicts increased lucency of the right hemithorax and a mass effect with a mediastinal shift to the left (Figure 1). The right lower lobe is largely replaced with emphysematous lung tissue. These findings reflected no changes from a previous chest X-ray from four years ago, when the patient presented to the ED complaining of a left chest wall pain and productive cough associated with clear sputum, reporting that after the cough, he subsequently heard a “pop” and began having left chest pain, worse with deep inspiration. A non-contrast CT of the chest revealed severe bullous emphysema involving the near entirety of the right lung (Figure 2A). The left lung has moderate paraseptal and mild centrilobular emphysema (Figure 2B).

The patient was ultimately diagnosed with COPD exacerbations, acute hypoxic respiratory failure, GBE, and amphetamine abuse. The patient was admitted, and medical management was initiated with low-flow oxygen, bronchodilators, a steroid taper, and empiric antibiotics. Considering that prior imaging studies have shown stable and unchanged findings, outpatient management by the pulmonologist was planned for monitoring the patient's severe emphysema. The patient’s symptoms improved with non-surgical management, and he was discharged after five days.

## Discussion

As stated previously, GBE is a progressive disease originally described by Burke [1] as characteristically presenting with severe progressive dyspnea with accompanying radiologic findings of extensive and predominantly asymmetric upper lobe bullous emphysema, typically seen in young, thin male smokers.

GBE poses a unique challenge in clinical practice due to its overlapping radiological findings with tension pneumothorax. In GBE, on plain radiographs, large asymmetrical bullae appear as a unilateral hyperlucency similar to the hyperlucency found in tension pneumothorax [6]. Additionally, GBE may exhibit a contralateral mediastinal shift similar to that of a tension pneumothorax [7-8]. Overcoming this diagnostic challenge is of utmost importance due to the significant difference in treatment approaches for GBE and pneumothorax. Pharmacologic and surgical methods are both employed in the management of symptomatic GBE. On the pharmacological front, treatment involves classic COPD management involving bronchodilators and inhaled glucocorticoids. For refractory cases of GBE and bullectomy, the surgical removal of one or multiple giant bullae may be indicated [9]. In contrast, managing a tension pneumothorax requires more immediate attention. In cases where the patient with tension pneumothorax exhibits hemodynamic instability, quick needle decompression followed by the insertion of a chest tube is necessary. However, if the patient is hemodynamically stable, there is a slightly extended timeframe to acquire radiographs before proceeding with needle decompression and chest tube placement [10]. In both situations, time is of the essence when handling a tension pneumothorax.

With the stark differences in their management, identifying a GBE from a tension pneumothorax is of utmost importance because needle decompression or insertion of a chest tube in a case of bullous emphysema can lead to pneumothorax and a bronchopleural fistula [2]. Thus, a previously medically manageable case can immediately turn into an inadvertently emergent scenario.

With that said, it is worth considering how a clinician approaches an acute scenario where both conditions are potential differential diagnoses. As stated previously, plain radiographs cannot provide definitive information between the two conditions. According to Venkategowda and Rao, the primary method for guiding the differential diagnosis is HRCT [11].

With HRCT, there is an invaluable sign to distinguish a GBE from a pneumothorax, as illustrated by Waitches et al.: the double-wall sign [12]. The presence of the double-wall sign is associated with the presence of a pneumothorax, whereas the absence of such a sign argues against the presence of a pneumothorax [12]. This sign is present when there is air outlining both sides of the bulla wall parallel to the chest wall [12]. In the case of a large bullous emphysema, as seen in Figure 2A, it may be difficult to differentiate it as a large bulla or a pneumothorax, but a careful review of multiple CT images, as demonstrated in Figure 2B, will show that the bulla wall is not parallel to the chest wall and no air is present in the pleura, arguing against a pneumothorax. The limiting factor here will be access to HRCT in an acute setting. For facilities that have limited access to HRCT, differential diagnosis will rely more heavily on patient history and the clinical picture.

## Conclusions

In this presented case of a 50-year-old male with severe bullous emphysema, we hope to have adequately highlighted the challenge of differentiating between GBE and tension pneumothorax in the acute setting. The treatments are vastly different between the two conditions, with GBE consisting mainly of conservative management and tension pneumothorax requiring invasive management. Differentiating between the two conditions is best achieved with HRCT. Additional research into the availability of HRCT in the acute setting may prove beneficial in identifying treatment gaps and disparities.
